# Entropy‐Mediated Nanoparticle Cellular Uptake

**DOI:** 10.1002/smsc.202300078

**Published:** 2023-11-27

**Authors:** Haixiao Wan, Duo Xu, Lijuan Gao, Li-Tang Yan

**Affiliations:** ^1^ State Key Laboratory of Chemical Engineering Department of Chemical Engineering Tsinghua University Beijing 100084 P. R. China

**Keywords:** biophysics, cellular uptake, entropy, entropy-controlled strategy, nanoparticle

## Abstract

Entropy, as one of the key parameters defining thermodynamic systems, is extensively explored in various scientific fields covering physics, chemistry, biology, etc. Particularly, understanding the entropic effects in cellular uptake is critical to understanding how physicochemical interactions govern the structure, response, and function of biological systems. Herein, the major types of entropy in biological systems, the underlying physical principles of their entropic forces, as well as the entropic effects emerging at the interfaces between nanoparticles and cell membrane in diverse biological processes, are introduced. The emphasis is on the physical principles arisen from entropy in the cellular uptake process. Finally, the remaining challenges and future directions for understanding and thereby manipulating entropy‐meditated nanoparticle cellular uptake are discussed. It is hoped that this review can explain some complicated and/or unique phenomena observed during cellular uptake, elucidate the physical mechanisms underlying their structures and dynamical processes, and advance entropy‐controlled strategy in the design and development of novel functional systems and materials toward advantageous biomedical applications.

## Graphical abstract

Entropy‐mediated cellular uptake points to new physical mechanisms underlying this important biological process and advances designing novel functional systems with advantageous biomedical applications. This article provides the first review regarding this topic.

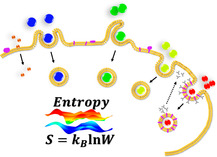

## 1 Introduction

Entropy not only forms the basis of contemporary thermodynamics, and statistical physics, but also has always been at the core of various discussions concerned with the evolution of the world, the course of time, and especially the enormous living processes in nature.^[^ ^1^ ^]^ As far back as 1943, Austrian physicist Erwin Schrödinger first proposed that physics and chemistry can interpret life phenomena in principle in his book What is Life,^[^ ^2^ ^]^ laying the foundation for molecular biology. In particular, he pointed out that life is dictated by negative entropy, which is the idea that living organisms reduce their own entropy by increasing the entropy of the outside world, to achieve a high degree of internal order. Thus, from the perspective of thermodynamics, the purpose of metabolism is to eliminate all the entropy that life inevitably generates to prevent the gradual decline toward equilibrium and ultimately death. Entropy has, however, risen in terms of the organism and the environment as a whole, which is in line with the second law of thermodynamics. Therefore, it is essential to focus on the fundamental contribution that entropy plays, to comprehend the fundamentals of living processes.

Being one of the most important components in living systems, biological lipid membranes have unique mechanical properties that are imperative for many biological processes, including cellular recognition,^[^ ^3^ ^]^ signal transduction,^[^ ^4^ ^]^ inter‐ and intra‐ cellular transport,^[^ ^5^ ^]^ and cell division.^[^ ^6^ ^]^ For example, because of the great diversity in the composition and structures of lipid membranes, maintaining the stability and function of cell membranes, especially under extreme conditions, has always been a great challenge. In nature, however, archaea can endure extremely harsh environments such as high salinity, high temperatures, acidity, or severe hypoxia without damage.^[^ ^7^ ^]^ In addition to its distinct metabolic process, entropic effect has been demonstrated to be essential to the maintenance of such a unique biological structure.[^7^, ^8^] Moreover, the entropic effect may also have an impact on the diffusion behavior of lipids or other molecules, the flexibility of lipids, and the viscosity of membranes.^[^ ^9^ ^]^ Therefore, without elucidating the contribution of entropic effect in biological systems, the mechanisms of lipid‐related biological events, particularly the cellular uptake, cannot be fully comprehended.

**Figure 1:**
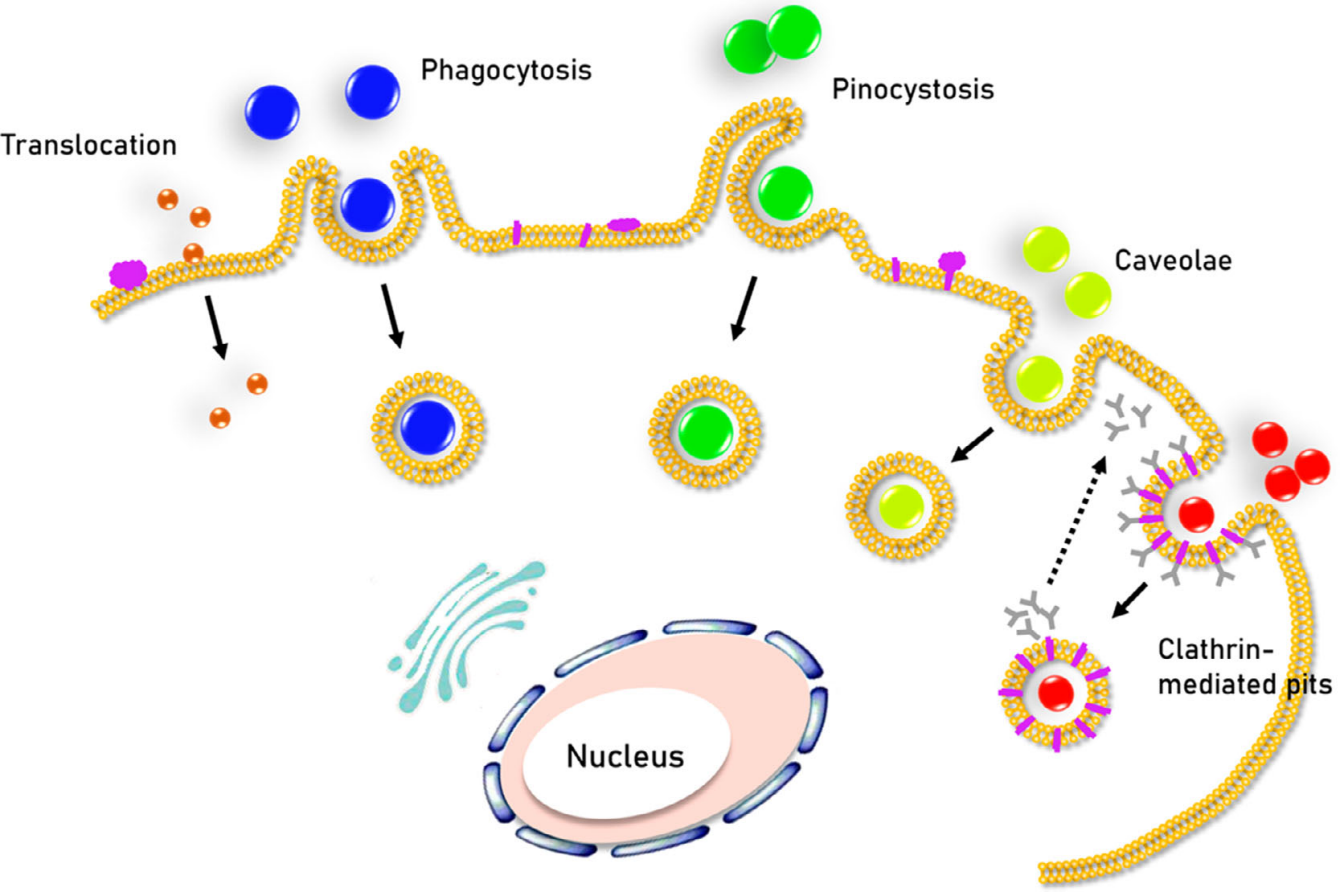
Schematic of the mechanisms for intracellular uptake of NPs.

**Figure 2:**
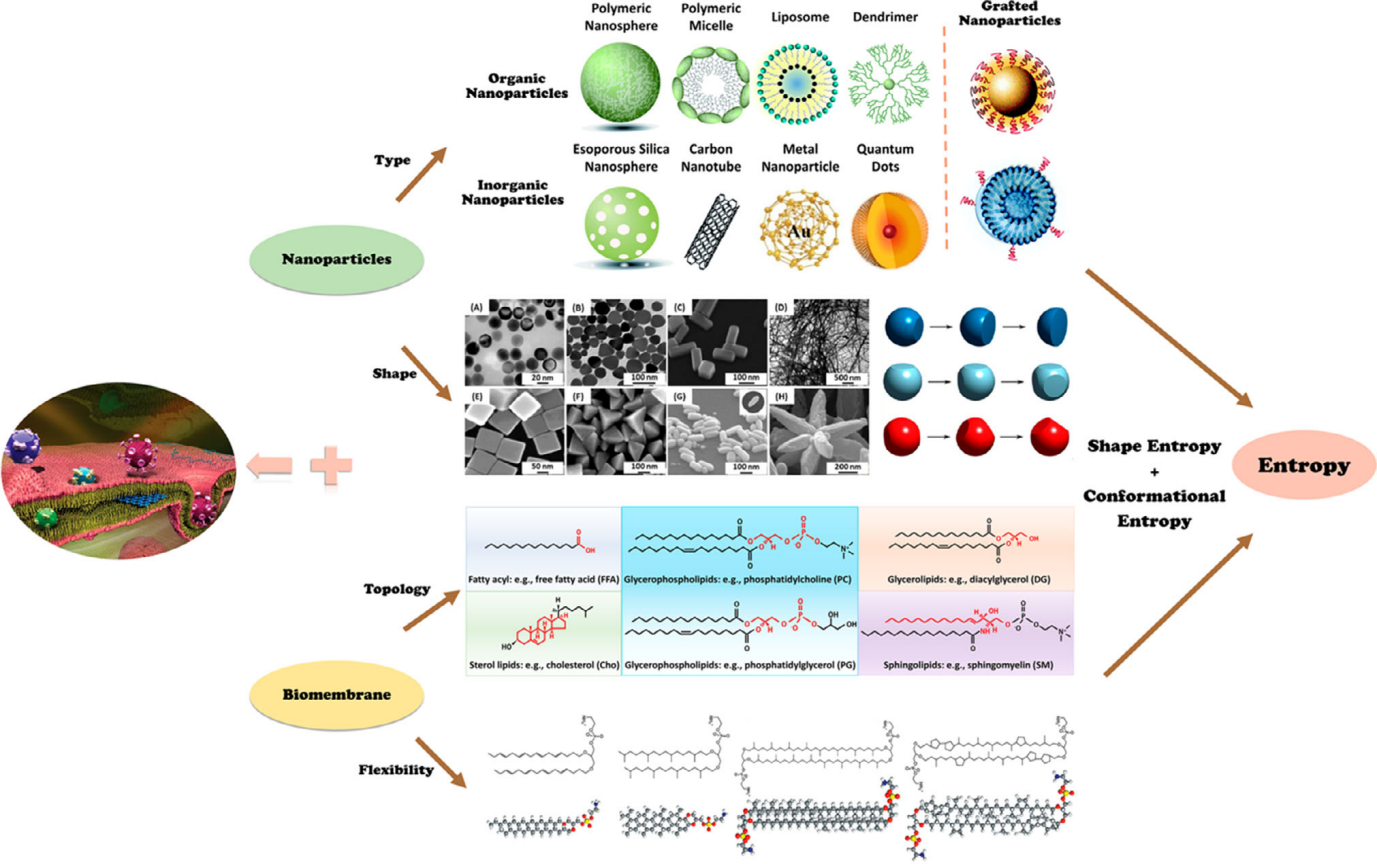
The schematic diagram of entropy‐mediated NP cellular uptake. The system's shape entropy and conformational entropy are significantly altered by the type and shape of NPs as well as by the topology and flexibility of the membrane, both of which have an impact on the processes of cell uptake. (Reproduced with permission.^[^ ^81^ ^]^ Copyright 2014, 2023, American Chemical Society, American Chemical Society).

On the other hand, the number of engineered nanoparticles (NPs) for various biomedical applications has grown tremendously over the last years due to advances in their synthesis and characterization.^[^ ^10^ ^]^ For most applications, the critical step is the transport through a cellular membrane. When NPs reach the exterior membrane of a cell, they can interact with the components of plasma membrane and then enter the cell.^[^ ^11^ ^]^ How do NPs fit in to the cellular uptake process? Experimental results indicate that engineered NPs, such as metal clusters, carbon nanotubes, fullerene, and quantum dots (QDs), could penetrate cell membranes and transport into cells of various types.^[^ ^12^ ^]^ In the schematic diagram illustrated in **Figure** **1**, the internalization processes of nanomaterials into cells through endocytosis are summarized. Micrometer‐sized particles can enter the cells through phagocytosis^[^ ^13^ ^]^ or pinocytosis.^[^ ^14^ ^]^ Caveolin‐dependent endocytosis involves the assembly of the hairpin‐like caveolin coats on the cytosolic side of the plasma membrane, forming a flask‐shaped caveolae of ≈50–80 nm in diameter.^[^ ^15^ ^]^ In clathrin‐mediated endocytosis, receptor–ligand binding triggers the recruitment and formation of “coated pits” (clathrin) on the cytosolic side of the plasma membrane.^[^ ^16^ ^]^ These pits self‐assemble into closed polygonal cages that facilitate the endocytosis. Clathrin assembly is also responsible for the formation of vesicle necking and the pinch‐off process in the late stage of membrane wrapping of NPs.^[^ ^17^ ^]^ In conclusion, different NPs, according to their size, shape, and surface properties, can enter into a cell via one of the endocytosis routes depicted above, where, fundamentally, entropic contribution may play a crucial role, but has been less well understood in contrast to its enthalpic counterpart. Simultaneously, the number of states is related to the intrinsic physical properties of the components, which determine the type of entropy and entropy effects. As a result, the entropy mechanism of cell uptake can be understood from the entropy effect induced by NPs and membranes. As shown in **Figure** **2**, there are two typical types of entropy that have a significant impact on the cellular uptake process. The first is the conformational entropy of phospholipid molecules in the cell membrane. The second is the shape entropy related to NP, which is an entropy effect generated from the geometric shape of the shape itself.

This review thereby provides a comprehensive description of the entropic contribution to the cellular uptake of diverse NPs by extracting and summarizing the relevant content from existed works. We first analyze the underlying physics of entropy‐mediated cellular uptake, from delineating the basic principles of entropy and entropic force in biological systems to elucidating the entropic effects in cellular uptake of NPs. Then, several aspects of entropy‐mediated cellular uptake are presented, including the entropy types explored, responsive behaviors mediated by entropy, relations of entropic effects to the architectures of biomacromolecules, as well as some key factors dictating entropic strategy in cellular uptake. Finally, we outline the remaining challenges and future directions for the entropy‐meditated NP cellular uptake. We hope this review will promote further efforts toward systematic research on the nature of entropy in various biological systems and the potential biomedical applications based on entropy‐controlled strategy.^[^ ^18^ ^]^

## 2 Underlying Physics of Entropy in Cellular Uptake

### 2.1 Entropy and Biological Systems

The concept of entropy first appeared in Clausius’ work explaining the second law of thermodynamics.^[^ ^19^ ^]^ He proposed that if a system underwent the quasistatic process from one equilibrium state to another, the entropy change dS between two equilibrium states can be defined as the heat transferred dQ divided by the thermodynamic temperature, *T*, that is, $dS=\frac{dQ}{T}$. Thus, the second law of thermodynamics states that any spontaneously occurring process will always lead to an increase in entropy, *S*.^[^ ^20^ ^]^ In simple words, the law explains that an isolated system's entropy will never decrease over time, that is, dS≥0, and thus entropy turns out to be one of the basic state variables in thermodynamics. Furthermore, in 1877, Boltzmann gave the relationship between entropy and the number of microscopic states of isolated systems, S∝lnW.^[^ ^21^ ^]^ It was later developed into $S=k_{B}\ln W$ by Planck,^[^ ^22^ ^]^ where $k_{B}$ is the Boltzmann constant. At this point, they made the connection between entropy and microscopic states as a measure of the uncertainty for an isolated system. Thus, the more states a system have, the more disorder there is. Consequently, an irreversible thermodynamic process tends to go from order to disorder, when the entropy reaches maximum. The second law is also known as the law of entropy increase, with which a spontaneous process takes places in the thermodynamic system only when it evolves from low entropy to high entropy.

Free energy, *F*, one of the essential variables in thermodynamics, is often determined by internal energy, *U*, *T*, and *S* as F=U–TS, where *U* represents the enthalpy, *T* is the absolute temperature, and *S* represents entropy.^[^ ^20^ ^]^ For hard matters, their structural organization is predominantly governed by the change in *U* due to the strong energetic interactions. In sharp contrast, for soft matters, including the biological systems, even a slight perturbation can cause considerable change in the structures owing to the significant contribution of entropy. More importantly, the entropic contribution to the free energy, with a general scale of a few $k_{B}T$ ($k_{B}T$is thermal energy), can overwhelm the interaction energy. In addition to the fact that macroscopic properties are governed by the microstructures, a deep insight into the entropic effect in the structural organization is essential to the development of novel materials. Thus, elucidating the relationship between entropy and order is particularly important for the biological systems.[^18^, ^23^]

Diverse entropy types can be identified from the biological systems, depending on the physical characteristics of the components, such as shape, surface compartmentalization, and mechanical properties, through which the components can present different degrees of freedom to remarkably increase the number of states.^[^ ^24^ ^]^ Briefly, the most common entropy in these systems should be the vibrational entropy which is directly related to the thermal fluctuation of every component. The rotational entropy and orientational entropy become the essential types of entropy when the components are anisotropic, as the anisotropy endows new degrees of freedom in the rotation and orientation, and the changes of them give rise to distinct states. For the components with certain shape under packing, the shape entropy can dominate their dense packing arrangement. When the surfaces of the components are tethered with chain‐like molecules, the conformational entropy originating from the chains will play a significant role in the structural organization of these systems.

### 2.2 Entropic Forces in Biological Systems

Entropy can drive the structural organization in a controllable way, through entopic forces which are distinct from the energetic interactions, such as van der Waals, hydrogen bonds, and π–π stacking. Entropic forces are effective forces that emerge from a system's statistical tendency to maximize entropy.^[^ ^25^ ^]^ That is, the origin of such a force is that the effect of thermal fluctuations tends to bring a thermodynamic system toward a macroscopic state corresponding to maximum entropy, that is, a maximum in the number of microscopic states that are compatible with this macroscopic state. One of the most important entropic forces in the biological systems is depletion force, referring to a type of attractive force that appears between larger suspended particles in a dilute solution containing smaller depletant solutes that tend to exclude themselves from the immediate surroundings of the larger particles.^[^ ^26^ ^]^ More generally, the large particle and depletant can include biomacromolecules, proteins, micelles, red blood cells, etc.^[^ ^27^ ^]^ Thus, this type of entropic force plays a crucial role in the structural organization of many fields, ranging from biomaterials to biophysics. Another important entropic force in biological systems is the elastic force due to the conformational entropy of the chain‐like molecules, such as DNA and polypeptides.^[^ ^28^ ^]^ Confining the conformational space of these molecules remarkably reduces the number of the states they can present.^[^ ^29^ ^]^ Thus, the statistical tendency to return to the maximal entropy translates into a macroscopic force, that is, the elastic force.^[^ ^30^ ^]^ This entropic force serves as a driving force in many systems containing biopolymers. Moreover, a standard example of an entropic force in the biological systems is the hydrophobic force. Although the hydrophobic force includes also a substantial enthalpic contribution, the largest part of this force in water comes from the entropy of the rearrangement of the 3D network of hydrogen bonds between water molecules.^[^ ^31^ ^]^ Hydrophobic force constitutes one of the most important types of nonspecific interactions in the structural organization of various biological systems, such as biological membranes.

### 2.3 Entropic Effects in Cellular Uptake of NPs

One of the most fundamental processes regulating biological activity is cellular uptake, which is governed by the properties of molecules, membranes, and their interactions.^[6a]^ As a consequence, the entropy‐mediated mechanism for the cellular uptake of NPs can be understood from the entropy effects caused by NPs and membranes, as illustrated in Figure 2. Thus, elucidating the entropy‐mediated mechanisms behind the lipid‐related biological events will provide fundamental foundation for the design and development of novel biomedical materials.

**Figure 3:**
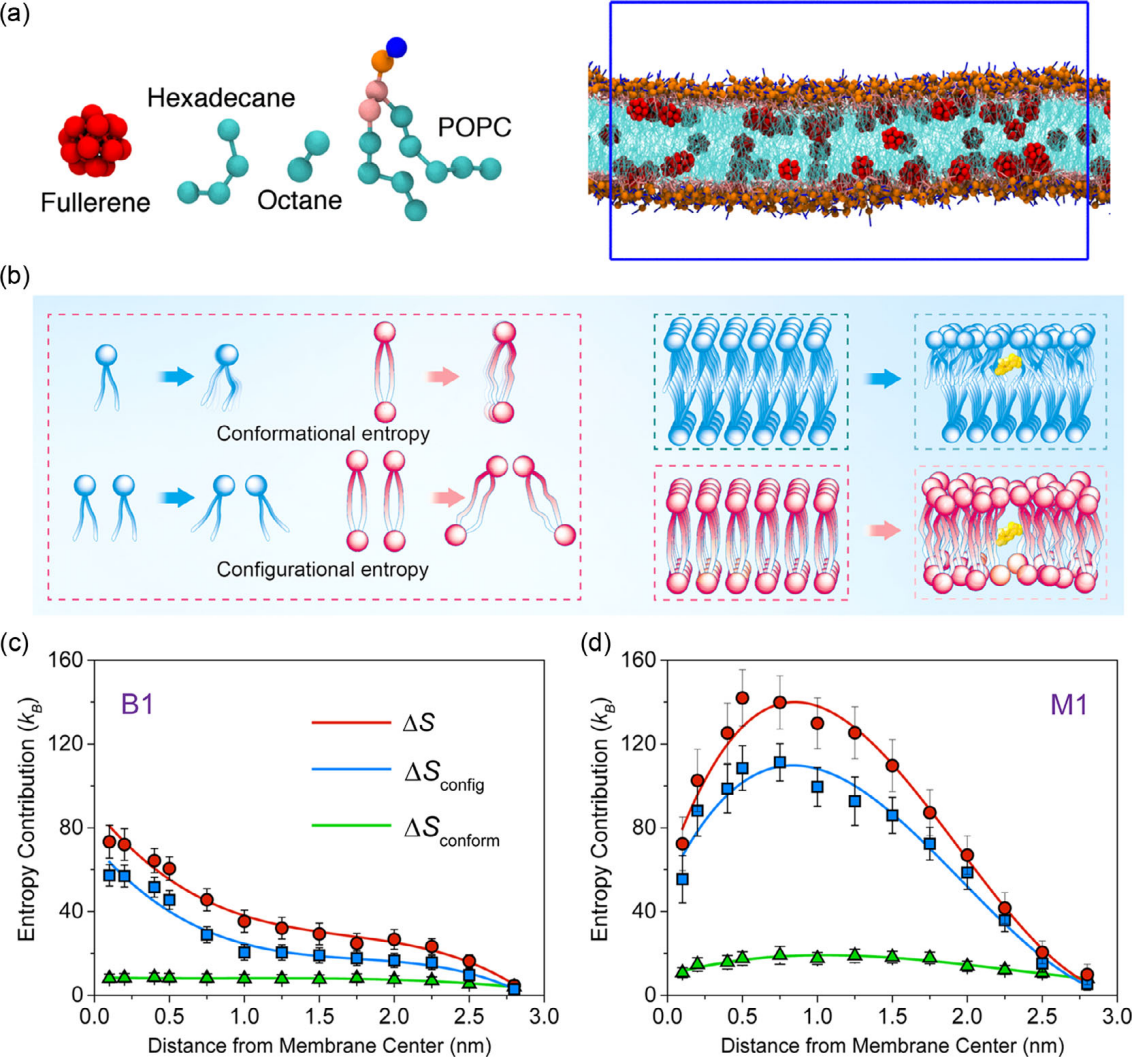
a) Snapshot of fullerenes in a palmitoyloleoyl‐phosphatidylcholine (POPC) membrane (Reproduced with permission.^[^ ^36^ ^]^ Copyright 2014 American Physical Society). b) Schematic illustration depicting the origin of conformational entropy and configurational entropy. Changes of the configurational entropy, conformational entropy, and total entropy of the system with membranes formed by c) monopolar lipid and d) tethered lipid. (Reproduced with permission.^[31f]^ Copyright 2023 American Chemical Society).

There are two types of entropy that have a substantial impact on the cellular uptake process. The first one is the conformational entropy of phospholipid molecules in cell membranes.^[28b]^ Diverse entropic effects can be produced due to conformational entropy, depending on how flexible and topologically diverse the phospholipids are.^[^ ^32^ ^]^ Furthermore, due to the interaction between NPs and membrane, the cell membrane will bend when it wraps NPs; thus, the space between the phospholipid molecules on one side of the bilayer enlarges, leading to greater conformational flexibility and a remarkably larger number of conformational states. Meanwhile, the gap between the phospholipid molecules on one side of the bilayer widens, increasing conformational space of one leaflet. The decrease in space on the other leaflet causes an imbalance between the two sides, producing a pronounced entropic effect that affects cellular uptake.^[^ ^33^ ^]^ Additionally, when grafted NPs or organic NPs, including polymer micelles, liposomes, dendrimers, and other soft NPs, interact with cell membranes, the conformational entropy from NPs also has a substantial impact on cell uptake.^[^ ^34^ ^]^ For instance, the cell membrane will become bent as a consequence of the interaction between the NP and the cell membrane. In this case, the NPs have a broader conformational space if it interacts with the sparse side of the phospholipid in comparison to the flat membrane because the contact area increases.^[^ ^35^ ^]^ However, once the interaction is on the opposite side, the conformational space of the NP is inhibited, leading to the emergence of a stronger entropic force that may inhibit the wrapping process. Furthermore, the location of NPs within the lipid membrane is greatly influenced by the conformational entropy of the lipid tails. For example, fullerenes are essentially insoluble in the majority of solvents, including alkanes. However, it has been demonstrated that C_60_ dissolves in the lipid bilayer, who shares alkanes’ intrinsic chemical characteristics (**Figure** **3**a).^[^ ^36^ ^]^ Theoretical research on the greater solubility of fullerene nanoclusters in lipid bilayers than in bulk alkanes reveals that the aggregation of fullerene is driven by entropy via dividing free energy into enthalpy and entropy components.^[^ ^36^ ^]^ Given the molecular structure of short chains, the structure and organization of the lipids in the membrane are impacted by both the configurational entropy caused by intermolecular arrangement and the conformational entropy caused by intramolecular deformation (such as molecular torsion and shape change) (Figure 3b).^[31f]^ Configurational entropy, which differs from conformational entropy, refers to the discrete representational locations or organizational structures of a system's constituent particles. It has been shown that the structural integrity and controllable permeability of cell membranes at high temperatures are primarily guided by the configurational entropy generated by distorted intermolecular organization of bipolar tethered lipids, using a combination of computer simulation and experiments (Figure 3c,d).

The second one is the shape entropy associated with NPs, an entropic effect that emerges from the geometry of the shape itself.^[^ ^37^ ^]^ Shape entropy will result in shape‐dependent directional entropy forces (DEFs) that maximize the state number of particles. For the systems of polyhedral packings, such a force is typically of the order of a few $k_{B}T$, making it comparable to depletion and van der Waals forces.^[38a]^ Therefore, it can govern particle phase behavior to self‐organize into a variety of ordered structures and finally maximize shape entropy in these systems by creating the shape‐oriented entropy force.^[^ ^38^ ^]^ Consequently, the shape entropy caused by various NP geometries, including spherical, cylindrical, square, hemispheric, and irregular shapes, as well as the synergistic effects of different NPs, can greatly influence the process of cellular uptake.

Other types of entropic effects, such as the orientation entropy caused by anisotropic NPs, may also have a significant influence on the processes and mechanisms of their cellular uptake. The broken symmetry of NPs prompts the emergence of orientation entropy, especially for nanorods or carbon nanotubes with well‐defined directionality.^[^ ^39^ ^]^ Additionally, the translational and vibrational entropy is correlated with the number of alternative configurations when molecules are involved in the biological structures with translational and vibrational modes.^[^ ^40^ ^]^ The fluctuation of the lipid membrane can also result in translational and vibrational entropy, which causes entropic pressure on two membranes with close separation and thereby determines the binding constants between the ligands and receptors anchored in the membranes.^[^ ^41^ ^]^

## 3 Entropy‐Mediated Interactions and Dynamics in Cellular Uptake

**Figure 4:**
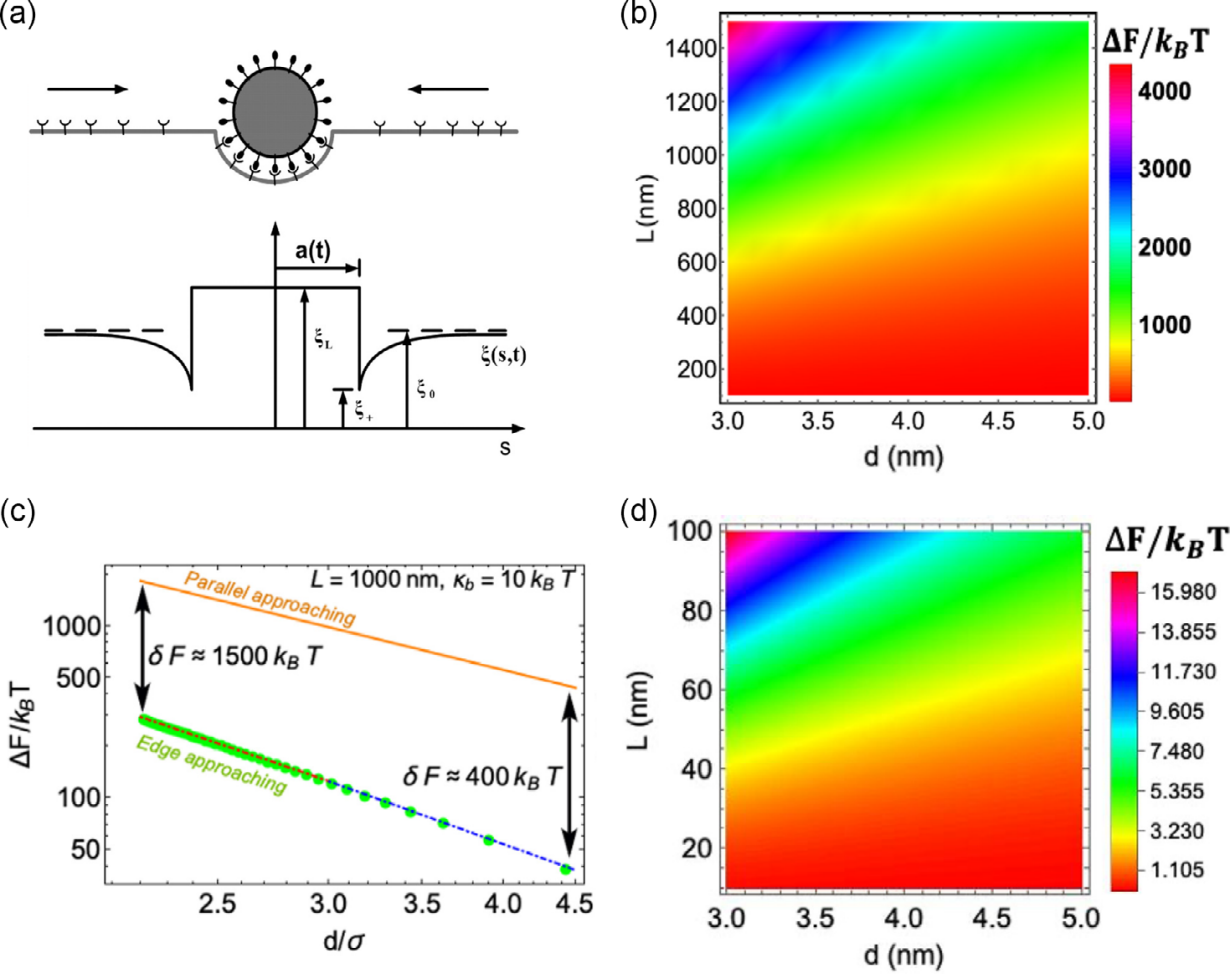
a) Diagram of a membrane with diffusive receptors enveloping an NP with ligands on its surface. (Reproduced with permission.^[^ ^44^ ^]^ Copyright 2005 National Academy of Sciences, U.S.A.). b) The cost of entropic energy to transport a 2D material. c) A comparison of a sheet's parallel and edge approaching modes. d) Small values of entropic energy cost for parallel approaching mode to a membrane. (Reproduced with permission.^[^ ^45^ ^]^ Copyright 2022 Elsevier).

The organization and evolution of the structures can be significantly influenced by the entropy effects in the cellular uptake of diverse biological systems. For instance, the attempt to molecularly construct an entropy‐driven 3D DNA amplifier (EDTD) capable of operating within living cells in response to a specific intracellular mRNA target is described. Because of the exclusive entropy‐driven force, mRNA target/EDTD interaction can precisely establish an autonomous DNA circuit inside living cells, allowing enormous signal amplification for ultrasensitive detection of the mRNA.^[^ ^42^ ^]^ Furthermore, many lipid or polypeptide macromolecules realize their function through the change of their conformation and/or spatial distribution, in which the effects of conformational and/or configurational entropy play a significant role.^[^ ^43^ ^]^ As shown in **Figure** **4**a, the receptor–ligand binding leads the membrane to locally curve around the NPs at the expense of higher elastic energy and reduced configurational entropy due to receptor immobilization.^[^ ^44^ ^]^ It was assumed that the receptors are distributed evenly over the cell membrane prior to particle contact, which is compatible with the state of maximum entropy. Once contact is established, the receptor density in the contact area rises to match the ligand density on the particle surface, which causes a reduction in the configurational entropy of the receptor and thereby mediates the following cellular uptake. Moreover, entropy forces from the conformational entropy of lipids are primarily in charge of controlling the interaction between 2D materials and cell membranes at its early stage of approaching (Figure 4b).^[^ ^45^ ^]^ In this case, the theoretical analysis with statistical mechanics and coarse‐grained (CG) molecular dynamics simulation of entropy energy barriers showed that micrometer‐sized graphene sheets prefer insertion into membranes that realize more fluctuations due to sharp angles, whereas nanoscale graphene sheets are more likely to adhere to the surface of cell membranes because of the relatively low entropic cost in comparison to the thermal energy of random Brownian motion (Figure 4c,d).

Clearly, it is essential to elucidate the effects of entropy on the process of cellular uptake. As stated earlier, the number of states in a specific space is evidently related to the intrinsic physical characteristics of the components, which determines entropy types and entropic effects, as illustrated in Figure 2. As a consequence, in this section we turn to systematically describing the entropic effects in the cellular uptake, based on the physical characteristics of the components, including flexibility, shape, and surface characteristics.

### 3.1 Flexibility

**Figure 5:**
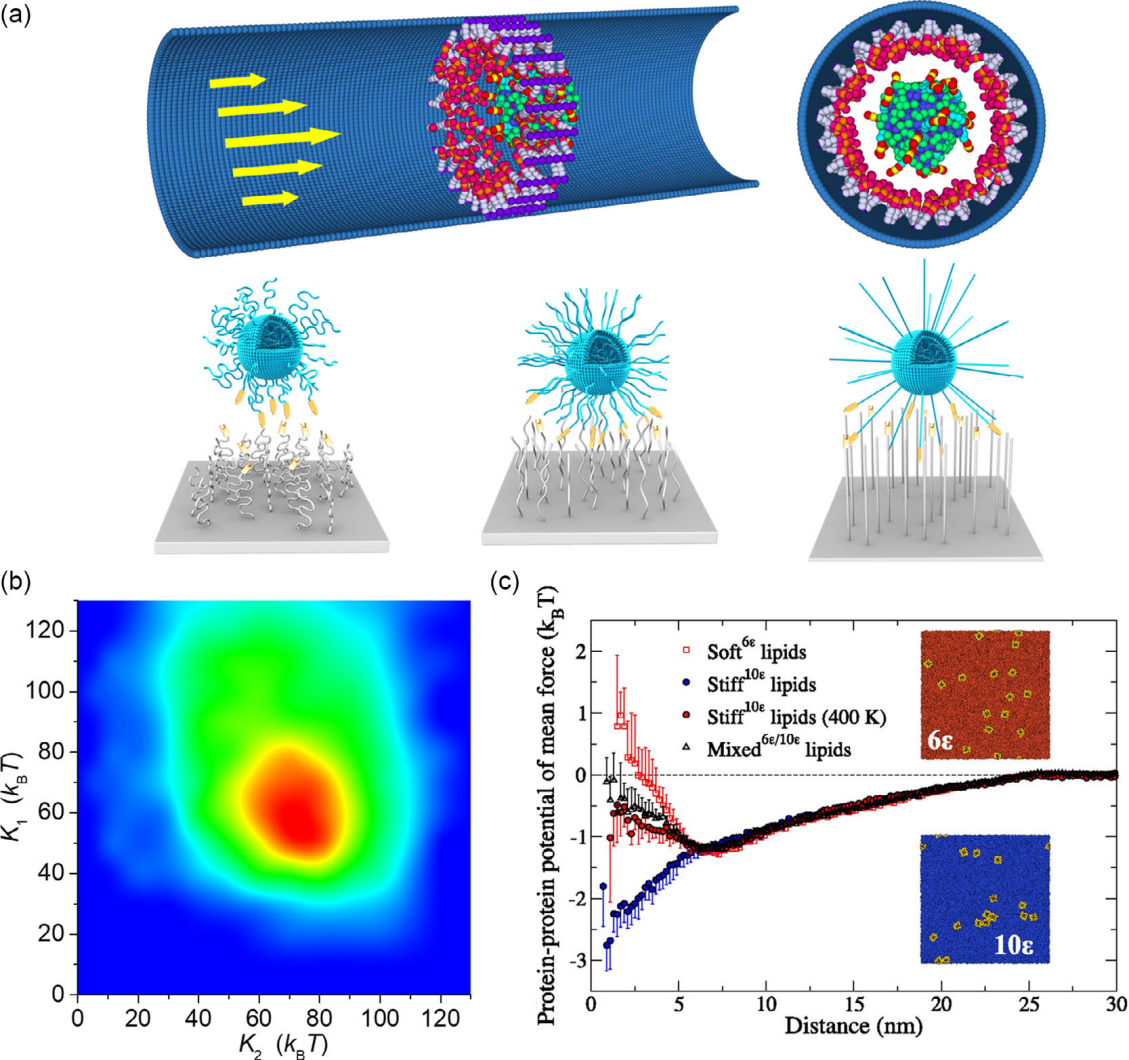
a) The representative dynamic regimes of flow‐induced transport of ligand‐functionalized vesicle and b) the vesicle‐capture efficiency with different chain flexibilities of ligands and receptor. (Reproduced with permission.^[^ ^48^ ^]^ Copyright 2019 The Royal Society of Chemistry) c) The estimated protein–protein potential of mean force for bilayer compositions of varying lipid flexibilities ranging from soft to stiff. (Reproduced with permission.^[^ ^49^ ^]^ Copyright 2018 National Academy of Sciences, U.S.A.).

We first focus on the entropy effect from the flexibility of molecular chains in cellular endocytosis. The flexibility of chain molecules stems from their enormous states of molecular conformation, which gives rise to conformational entropy.^[^ ^35^, ^46^ ^]^ On the other hand, ligand–receptor binding plays a crucial role in relevant biomedical functions and applications, such as targeted drug delivery vectors and biosensors.^[^ ^47^ ^]^ To optimize ligand–receptor binding and selectivity for the ligand‐functionalized vesicles undergoing nanofluidic transportation, the intrinsic physical properties of ligands and receptors, particularly the chain flexibility, were systematically examined (**Figure** **5**a).^[^ ^48^ ^]^ The results demonstrate that there are optimal chain flexibility and vesicle rigidity to achieve the maximum ligand–receptor binding and capture probability, exhibiting a nonmonotonic dependence on their intrinsic properties (Figure 5b). A theoretical model of the blob theory was further developed to analyze the physical mechanism of the ligand–receptor binding in this process, which indicates that the superentropic effect of semi‐flexible chains is the fundamental factor for optimizing ligand–receptor binding efficiency. The findings provide guidance for the design of drug delivery systems that can keep transport via confined spaces, including blood capillaries or microfluidic devices in tissue engineering for improved targeting accuracy. Additionally, the lipid flexibility determines the clustering and spatial localization of proteins in lipid membranes, which can affect the function of membrane proteins in some biological processes, including endocytosis and budding.^[^ ^49^ ^]^ Changing the flexibility of the individual lipids will alter the entropic force in essence. Indeed, the CG simulations showed that the entropic force can result in various influenza A matrix 2 (M2) assembly geometries for the lipids with different flexibilities. M2 proteins do not cluster when they are embedded in a soft membrane, but instead “bounce off” one another (Figure 5c). In this case, the force to drive protein clusters is weaker than one unit of thermal energy. When M2 is inserted into the membrane made up of stiff lipids, however, stable protein clusters spontaneously appear, and the formation of such protein clusters is reversible. The revealed results offer theoretical and conceptual guidance for virion release from the infected host cell, in which the entropic forces control the formation of M2 cluster regulated by membrane lipid flexibility.

**Figure 6:**
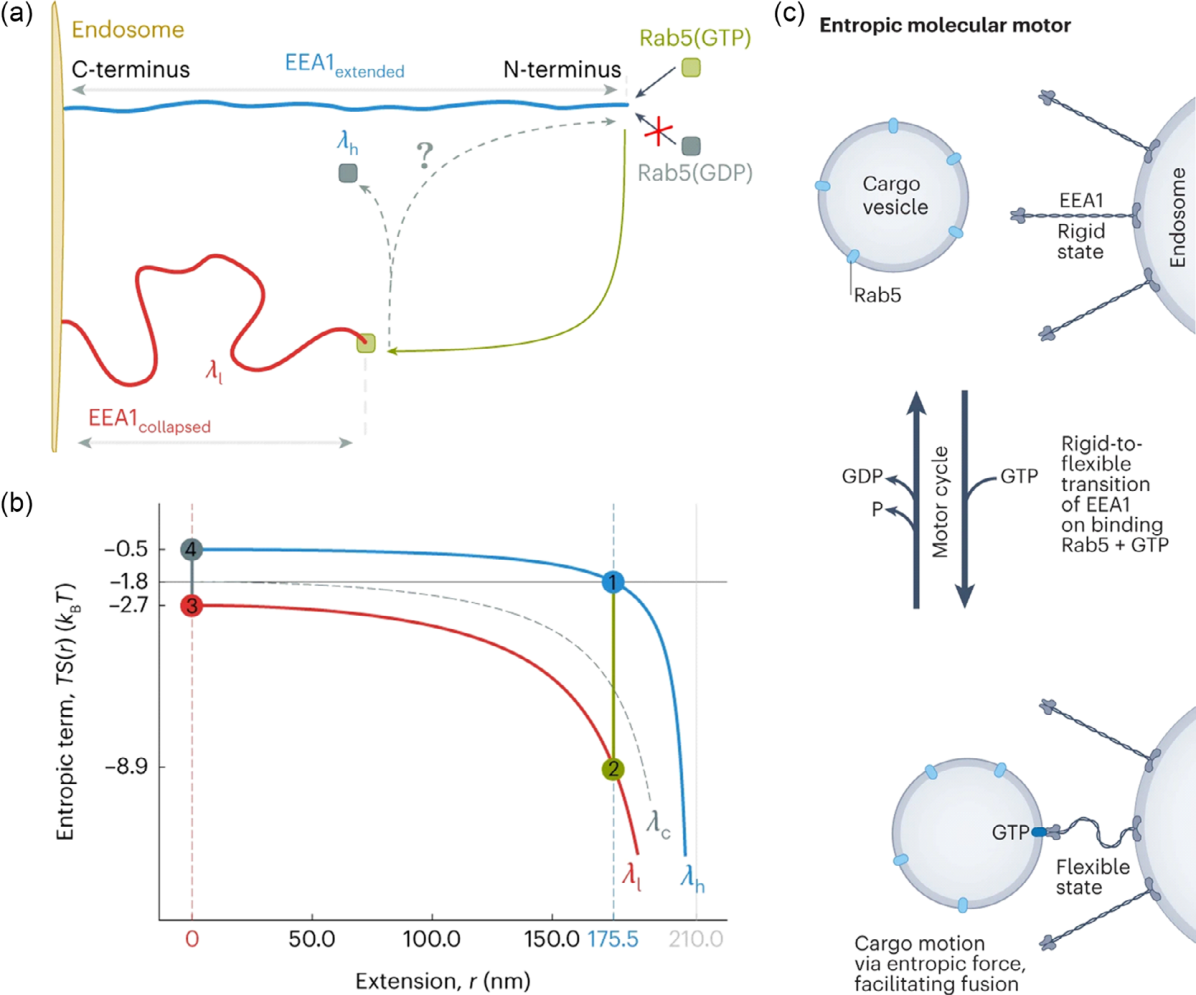
a) Schematic illustration and b) conformational entropy of the collapsed/extended states of EEA1 on binding/unbinding to the active/inactive forms of Rab5. (Reproduced with permission.^[51b]^ Copyright 2023 Springer Nature.) c) EEA1–Rab5 “entropic” motor switches the coiled‐coil protein EEA1 from a rigid to flexible state upon binding to Rab5. (Reproduced with permission.^[^ ^82^ ^]^ Copyright 2023 Springer Nature).

The development in entropy‐controlled strategy has been focused on the paradigm of equilibrium thermodynamics: maximizing entropy while minimizing free energy.^[^ ^18^ ^]^ Understanding the entropic effect in biological activities with thermodynamic nonequilibrium, however, remains one of the great challenges. Entropy may actually be more important for a number of biological activities in the nonequilibrium situations. For example, the cytoskeletal cargo transporters known as motors use chemical energy to produce power strokes to travel along actin filaments or microtubules, which has been well studied as the most general case for the intracellular delivery. However, the recent works have demonstrated a new mechanism to realize this goal, based on, interestingly, the entropic motor with two components, EEA1 and Rab5, driven by a GTPase cycle.^[^ ^50^ ^]^ Specifically, when EEA1 binds to GTPase Rab5, its conformation often shifts from a more rigid “extended” state to a more flexible “collapsed” state (**Figure** **6**a). Entropic effect thereby drives the EEA1 to collapse or adopt reduced end‐to‐end distance configurations as a result of its flexibility transition, which essentially provides an entropy force that pulls vesicles close to the proximity of the endosome, increasing the probability of membrane fusion (Figure 6b,c). This points to a new type of motors, that are regulated by entropy, in the biological systems, aiding in moving materials within and outside of cells.

### 3.2 Shape

**Figure 7:**
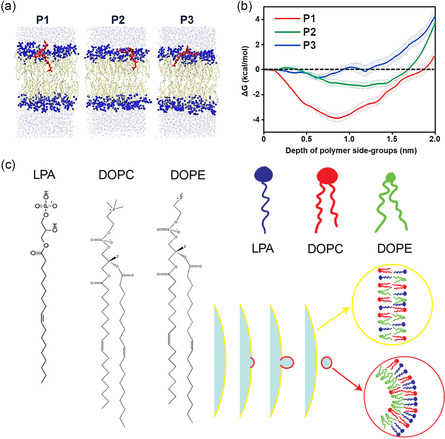
a) Representative configurations of the P1–P3 polymers (red) interacting with the phospholipid bilayers and b) corresponding free‐energy profiles as a function of the depth of the polymer side chains. (Reproduced with permission.^[^ ^57^ ^]^ Copyright 2021 American Chemical Society). c) Lipid sorting based on spontaneous curvature consisting of lipids with various shapes, such as inverted‐conical for Lyso‐Phosphatidic acid (LPA), cylindrical for unsaturated 1,2‐dioleoyl‐sn‐glycero‐3‐phosphocholine (DOPC), and conical for 1,2‐dioleoyl‐sn‐glycero‐3‐phosphoethanolamine (DOPE). (Reproduced with permission.[^24^, ^83^] Copyright 2004, 2022, 2020 Wiley, Elsevier, American Chemical Society).

A key characteristic of NPs is their shape, which has been proved to exert a significant impact on biological processes, such as particle adhesion, distribution, and cell uptake.^[^ ^51^ ^]^ For example, ellipsoidal particles are less favorable to be internalized by the cell membrane than spherical ones due to their larger local curvature than sphere‐shaped ones (resulting in higher bending energy).^[^ ^52^ ^]^ The correlation between the translational and rotational diffusion of rod‐shaped NPs on a lipid membrane was investigated using polarization‐resolved dark‐field optical microscopy. Large‐step scanning on the lipid membrane occurs as a result of the conformational entropy release, which is typically linked to the encouragement of translational diffusion.^[^ ^53^ ^]^ The endocytosis process of spherocylindrical NPs that are initially upright docking on the membrane plane involves lying down and subsequently standing up.^[^ ^54^ ^]^ Free energy analysis reveals that the shape of NPs disrupts the symmetry of the curvature energy pattern, which determines the endocytosis pathway and entry angle. The impact of shape can, fundamentally, be attributed to the entropic effect due to shape entropy, which operates through DEFs.^[^ ^55^ ^]^ This entropic effect can be regulated by the degree of anisotropy of particles. In general, particles with more pronounced anisotropy tend to have stronger DEFs, making them more prone to large shape entropy.[^24^, ^57^] By examining the shape complementarity of various proteins, it was discovered that the conformation of the lipid chain between two proteins is greatly restricted when two proteins with shape complementarity approach one another.^[^ ^56^ ^]^ Hence, lipid molecules have a tendency to move out of the space between the two proteins to maximize the entropy, which generates entropic effect inducing attraction between proteins with complementary shapes, enhancing the protein aggregation. Recently, experiments and all‐atom simulations were also employed to investigate how polymer side chains with different molecular shapes (“needles” and “razors”) contribute to interactions between bacterial bilayers and polymer structures (**Figure** **7**a).^[^ ^57^ ^]^ The potential of the mean force was calculated to assess the free energy, which indicates that the insertion of the polymer side chains inevitably enhances the entropy of the system (Figure 7b). The penetration is an exergonic process that is favored by entropy but opposed by enthalpy. In other words, “antibacterial” features of these polymers result from an entropy‐driven spontaneous contact between their side chains and the bacterial lipid bilayer.

Although the fundamental physics of lipid sorting under thermal equilibrium is driven by the trade‐off between bending energy, mixing entropy, and interactions between species, the distribution of lipids can be homogenized by mixing entropy, as demonstrated by experimental and theoretical investigations (Figure 7c).^[^ ^58^ ^]^ A lipid with a cone shape called cardiolipin (CL) is mostly found in the curved membranes of bacteria and mitochondrial cristae.^[^ ^59^ ^]^ It has been proposed that this particular localization is entropy driven because the entropic effect from the conical shape of the CL reduces curvature frustration. It was also discovered that, due to the shape entropy, the stable endocytosis state for rod‐shaped particles has a small but high fraction of wrapping; increasing the aspect ratio is not helpful for complete wrapping.^[^ ^60^ ^]^ In this case, particles enter through the submarine mode with their long edges parallel to the membrane for high aspect ratios and circular tips, while particles enter the tip first through rocket mode for small aspect ratios and flat tips.

### 3.3 Surface Modification

Surface modification, which can achieve functionalization, tailor surface reactivity, or increase stability, is a crucial manipulation for NP applications. Hydrophilicity/hydrophobicity, charge sign/density, and specialized interactions (such as receptor–ligand interactions) are the primary categories of surface features.^[^ ^61^ ^]^ Through anisotropic chemical modification of homogeneous surfaces, directionality can be introduced into the interactions between components. Superentropic effects, however, might result from interactions between components that are influenced by these surface features. On the one hand, the presence of surface‐directed interactions induces equivalent limit for entropic effects, so that the entropy of the system can only be maximized under the restrictions of the surface contacts, as opposed to the pure entropy system without energy contribution at all.^[^ ^62^ ^]^ The existence of surface orientation, on the other hand, occasionally acts as a platform for the entropic effect, allowing the creation of entropic forces to work on this platform. It should be emphasized that if there are some ligand modifications on the surface of NPs, the characteristics of the new surface (i.e., ligand) will be far more significant than that of the old surface (i.e., NP core).

**Figure 8:**
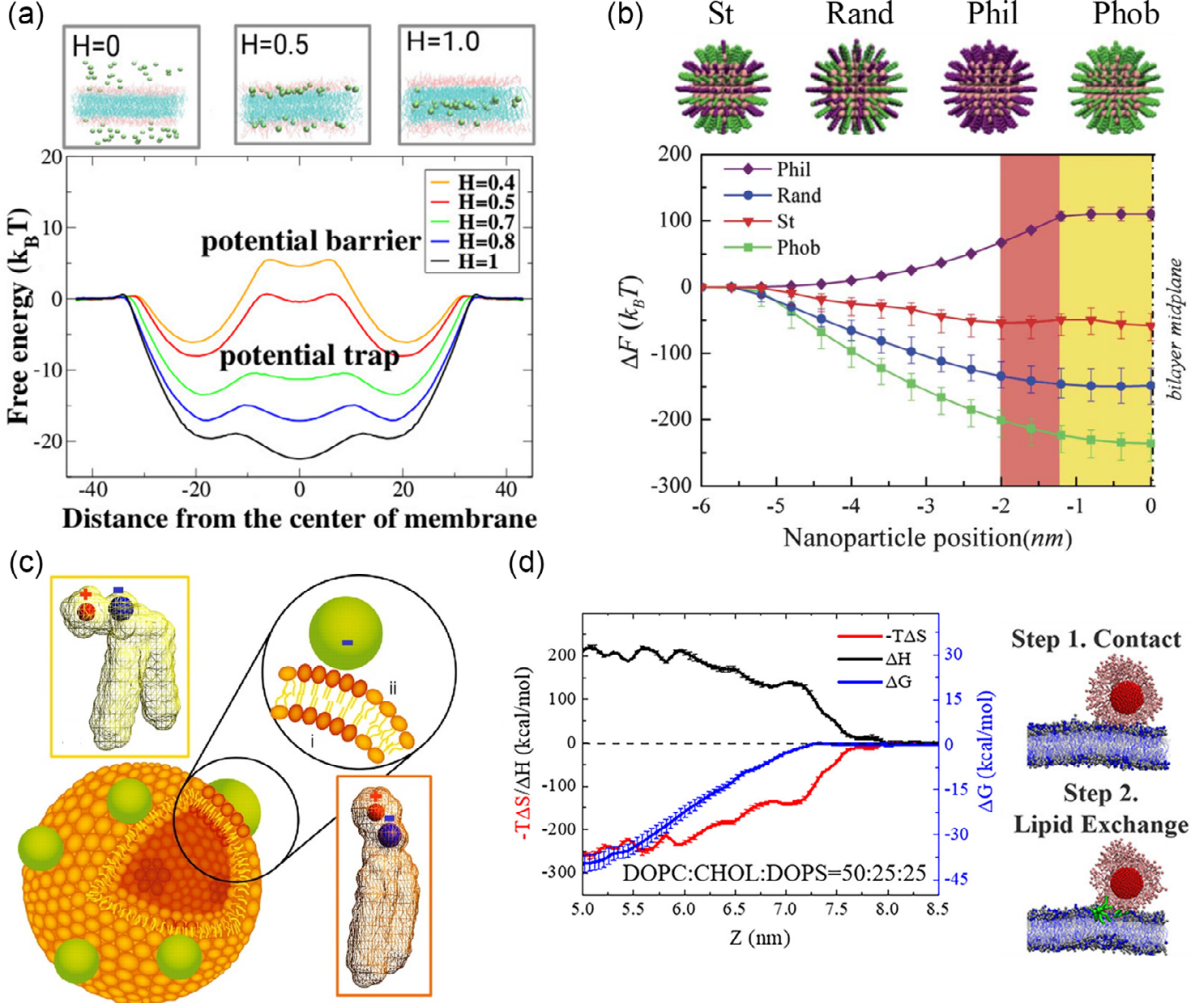
a) Free energy profiles as a function of the distance from the membrane center at different hydrophobicities, H. (Reproduced with permission.^[^ ^64^ ^]^ Copyright 2017 American Chemical Society). b) Free energy changes associated with the penetration of various types of ligand‐coated NPs as a function of the distance between the NPs center and bilayer midplane. (Reproduced with permission.^[^ ^66^ ^]^ Copyright 2012 The Royal Society of Chemistry). c) Schematic diagram of phospholipid bilayer vesicles with binding NPs. The combination of anionic NPs with lipid bilayer in the fluid phase results in the NPs templating the gel phase where the NPs are combined. (Reproduced with permission.^[^ ^70^ ^]^ Copyright 2008 National Academy of Sciences, U.S.A.). d) Enthalpy and entropy changes during the interactions. (Reproduced with permission.^[^ ^76^ ^]^ Copyright 2022 American Chemical Society).

Hydrophobic interactions, that belong to entropic effect in nature,^[^ ^31^ ^]^ cause self‐organized phospholipid molecules to create amphiphilic cell bilayers, which are isolated from aqueous solutions by their hydrophobic tails. Because of this, the hydrophobicity of NPs is a crucial factor affecting how they interact with a membrane.^[^ ^63^ ^]^ The translocation of NPs with variable levels of hydrophobicity through lipid bilayer membranes was examined using CG molecular dynamics simulations.^[^ ^64^ ^]^ The average escape rate of NPs can be obtained via mapping the translocation rate through computing the free energy map, and the maximum translocation value was mapped to the maximally flat free energy landscape (**Figure** **8**a). The findings of both the average first‐passage time of diffusion particles in the potential of mean force and the passive translocation of NPs obtained from the simulation demonstrated that a narrow window with significant NP translocation rates occurs and the optimal hydrophobicity is close to *H* = 0.5, where *H* denotes hydrophobicity of NPs. The corresponding free energy landscape is maximally flat, and the remaining barrier can be attributed to the entropy decrease of the tails caused by particle accommodation. The latter has only a few orders of magnitude of *k*_B_T, which explains why translocation rates are so high. It has successfully synthesized a novel riboflavin (Rf)‐based ligand by conjugating Rf with citric acid. Thermodynamic parameters of the binding process reveal an energetically favorable binding driven by entropy changes. The modification at the ribityl chain of the Rf molecule reduces hydrophilicity of the structure, causing displacement of water molecules from the hydrophobic pocket of RCP upon binding and raising the entropy of the system.^[^ ^65^ ^]^

Furthermore, dissipative particle dynamics simulations were employed to examine how single‐layer‐covered NPs with various types of surface ligands penetrate cells.^[^ ^66^ ^]^ Four distinct ligand modalities were taken into account: NPs coated with homogeneous hydrophilic or hydrophobic ligands, NPs with randomly mixed ligands with the same hydrophilic‐to‐hydrophobic ratio, and stripe NPs with alternating hydrophilic and hydrophobic groups (Figure 8b). The thermodynamic integration approach was implemented to determine the free energy distribution of ligand‐coated NPs that penetrate the lipid bilayer. According to the free energy analysis, striated NPs experience the lowest‐energy barrier during transmembrane transport among the four ligand modes examined. This consequence can be related to the entropy loss of striped NPs as a result of its constrained rotation via the anisotropic ligand pattern in the bilayer. The translocation free energy profile is significantly flattened and permeation is drastically increased; upon that the surface of NPs changes from having a random, heterogeneous distribution of hydrophobic and hydrophilic areas to even, homogeneous patterns. The improved lipid bilayer permeability caused by the adsorption of NPs with adjustable hydrophobicity was demonstrated by the Monte Carlo simulation with explicit solvents.^[^ ^67^ ^]^ The adsorption transition of NPs on the bilayer occurs depending on the range of relative hydrophobicity. Within a certain range, NPs can translocate through the bilayer, irreversibly disrupting its structure, causing higher permeability of water and small solutes.

Additionally, the effect of the surface charge of NPs can also be quite eminent. Especially, the sign and spatial distribution of surface charges have a remarkably impact on their diffusion behavior and directional transport.^[^ ^68^ ^]^ Randomly charged NPs go through directional superdiffusion transport instead of the Fickian diffusion experienced by neutral NPs. However, due to lipid membrane fluctuation and surface reconstruction caused by electrostatics, the dynamics of evenly charged NPs dramatically improve Fickian diffusion.^[^ ^69^ ^]^ The results have extensive implications for how we understand and control charged NPs transport across cell membranes. A single‐component phospholipid bilayer containing a choline phosphate head group can undergo surface reformation as a result of the nonspecific adsorption of charged NPs (Figure 8c).^[^ ^70^ ^]^ Positively charged NPs cause localized fluidization of the initially gelatinous membrane, while negatively charged NPs cause local el in other fluid bilayers. When these NPs combine, hydrated water is released by the phosphocholine head group, which may be responsible for the entropy loss produced by gel and partially responsible for the considerable contribution of entropy, as shown by the evident discrepancy between enthalpy and free energy.

Hydrophobic and electrostatic interactions, coupled with the increase in entropy induced by the protein unfolding, are the main driving forces for protein adsorption.^[^ ^71^ ^]^ Before the NPs approach the membrane surface, such as in the blood, plasma, and interstitial fluid, where there is a large amount of proteins, the protein corona of NPs will be formed, which may interfere with the cellular uptake of NPs.^[^ ^72^ ^]^ In some cases, the formation of protein corona can potentially inhibit cellular uptake. For instance, serum proteins adsorbed on NPs can prevent the cellular uptake of HeLa cells.^[^ ^73^ ^]^ Experiments revealed that the occurrence of protein crowns may cause functional NPs with proteins to lose some of their targeting specificity when exposed to biological conditions.^[^ ^74^ ^]^ Biomolecules and membranes are severely and uncontrollably impacted by these unmodified NPs. By encasing the core in a biocompatible shell comprising, for instance, lipids or a polymer brush, it is possible to effectively screen and inhibit such nonspecific ionic, dipole, or entropic attractive interactions.^[^ ^75^ ^]^ A detailed investigation of the interactions between cationic lipid NPs (cLNPs) and several model cell membranes revealed that the binding of cLNPS to membranes is an entropy‐driven process controlled by a two‐step mechanism (Figure 8d).^[^ ^76^ ^]^ More important lipid exchange (the separation of cLNPS‐covered lipids and subsequent flipping and embedding into the membrane) follows direct contact to start the process. Therefore, cLNPS can be utilized to distinguish membranes with different lipid components, such as the outer and inner membranes of bacteria and red blood cell membranes. The complex entropy–enthalpy competition that occurs when cLNPS interacts with the membrane was therefore found to be capable of driving the membrane‐targeting behavior of cLNPS.

### 3.4 Entropic Effects Contributed by Small Molecules

The regulation of the transport of small molecules across the membrane is one of the primary roles of the membrane. While the membrane transport, as a rule, involves unique channel forming peptides and proteins, various small, uncharged molecules, such as O_2_, CO_2_, water, NO, CO, etc., can penetrate into the cell membrane via simple diffusion. Simple diffusion is a passive process by which molecules move across the membrane, driven by a concentration or an electric potential gradient, without the aid of an intermediary such as a membrane protein.^[^ ^77^ ^]^

The transport of small molecules requires that they are at least slightly soluble in the lipid bilayer. The hydrophobic effect has traditionally been explained in terms of the ordering of water around a hydrophobic solute. Therefore, the partitioning of a hydrophobic solute from water into a bulk hydrophobic solvent is typically driven by a strongly favorable entropy component and a small enthalpy term, with a large, negative heat capacity change.^[^ ^78^ ^]^ Atomistic computer simulations of hexane in a dioleoyl phosphatidylcholine model membrane were conducted to acquire a better understanding of how a small molecule partitions into the lipid bilayer. The results demonstrate that hexane preferentially partitions to the center of bilayer, which is nearly exclusively driven by a favorable entropy change, compatible with the hydrophobic effect. Partitioning to the densest region of the acyl chains, however, is dominated by a favorable enthalpy change with a small entropy change, corresponding with the “nonclassical” hydrophobic effect or “bilayer” effect.^[41a]^ Moreover, the insertion of anionic ibuprofen into the lipid bilayer is a thermodynamically spontaneous and entropy‐driven process starting from the pure aqueous phase by analyzing the thermodynamic factor. Entropy changes relative to the reference state (associated with increased freedom of motion and with an increased number of possible configurations) are stabilized in this process. Two factors may be utilized to explain the increase in the number of possible microstates: 1) the presence of ibuprofen increases the dynamic possibility of phospholipid chains (or, equivalently, decreases the dynamic structural order) and 2) the absence of ibuprofen in the aqueous phase increases the degree of freedom in the dynamic structure of water.^[^ ^79^ ^]^ Furthermore, the direct interaction of di‐mannose with membrane has a short residence time, with di‐mannose exhibiting a small free energy bias toward the water–phospholipid interface, and this bias is driven by maximizing the water entropy of the whole system.^[^ ^80^ ^]^

## 4 Summary and Perspective

A fundamental understanding of the role of entropy in the physicochemical interactions of cellular uptake is critical because it regulates various biophysical phenomena and processes, resulting in more effective and secure biomedical applications of nanomaterials. In this review, we first introduce several types of entropy in biological systems, the underlying physical principles of their entropic force, as well as the entropic effect emerging at the interfaces between NPs and cell membrane in diverse biological processes. The emphasis is on how to precisely regulate and design the cell uptake process through exploiting various types of entropic effects.

Comprehensive efforts have been made in this important and promising field, yet there are still many challenging issues. Although it is clear that the entropic effects during cell uptake can significantly impact the organization and evolution of the structure, the entropy types involved in such complex biophysical processes and the phenomena governed by entropy require further exploration, which is beneficial for us to better understand the potential mechanisms of these activities. In addition, it has become increasingly effortless to manipulate and modify the physical and chemical properties of bulk materials on a large scale to fulfill particular biological purposes. However, the creation of a “perfect” nanodelivery system is far from being achieved. To this end, further development of design principles based on the entropy‐controlled strategy revealed in biological systems provides an alternative way to promote tailored structures and functionalities of innovative materials with promising biomedical applications.

To fully elucidate the entropic effects involved in the cellular uptake of NPs, it is vital to comprehend them from the perspectives of all‐atom, meso, and even macroscales. Thus, it is useful to develop hybrid simulation methods that combine CG modeling with all‐atom simulation. Additionally, it is essential to improve current force fields or create new force fields to more accurately mimic physiological conditions when computing interactions and dynamics at the interfaces between NPs and biomolecules (particularly particular proteins) inside and outside the cell membrane. To achieve direct computational design, theoretical verification, and experimental interpretation, it is critical to integrate simulation and experiment more closely. These computational advances in this field, on the other hand, provide essential insights into the molecular mechanisms of the cellular uptake that are difficult to obtain through experimentation. Therefore, developing or modifying experimental approaches is indeed necessary to validate the computational findings regarding the entropic effects in NP cellular uptakes.

## Conflict of Interest

The authors declare no conflict of interest.
